# Breast and Ovarian Cancer Risk due to Prevalence of *BRCA1* and *BRCA2* Variants in Pakistani Population: A Pakistani Database Report

**DOI:** 10.1155/2011/632870

**Published:** 2011-03-24

**Authors:** Ayesha Farooq, Abdul Khaliq Naveed, Zahid Azeem, Tausif Ahmad

**Affiliations:** Department of Biochemistry and Molecular Biology, National University of Sciences and Technology, Rawalpindi 46000, Pakistan

## Abstract

*Introduction*. Pakistani population has a very rich anthrogeneological background with waves of migration from neighboring regions. Incidence rates of breast and ovarian cancer in Pakistan are on such a rapid rise that it is necessary to check the contributory factors, genetic and nongenetic. An insight into the prevalence data emphasizes the formulation of a *BRCA1* and *BRCA2* database for the Pakistani population. *Method*. In this study conducted by authors, data from diagnosed cases of both sporadic and inherited female breast and ovarian cancer cases was gathered after performing molecular genetic analysis by screening for alterations in the coding sequence of the *BRCA* gene. The region of interest was analyzed by the aid of various molecular biology tools such as automated DNA sequencer. Bioinformatics software was used to interpret the results, and database was prepared. *Results*. Mutational screening of the exons in all the samples of our study group did not reveal any pathogenic mutation. These results along with the results of the previous Pakistani studies for both *BRCA1* and *BRCA2* genes were summed up to prepare a Pakistani database. Percentage involvement of these genes was estimated. Nine percent of these cancers show alterations in *BRCA1* gene while 3 percent have shown *BRCA2* variants. The remaining 88 percent of breast and ovarian cancers can be attributed to the involvement of other genes.

## 1. Introduction

 Breast cancer is the most prevalent cancer in the world, and ovarian cancer is the sixth most common cancer in the world [1]. Inherited mutations in the breast cancer susceptibility gene 1 (*BRCA1*) [MIM 113705] and breast cancer susceptibility gene 2 (*BRCA2*) [MIM 600185] are associated with a high risk of developing breast and ovarian cancers in females of different age and ethnic groups. These well-defined high-penetrance genes show loss-of-full function germ line mutations in hereditary cases and decreased expression in sporadic tumors [2–4]. Approximately, 5 to 10 percent of breast cancer [5] and at least 10 percent of ovarian cancers [6] are hereditary. Sequence variation in the *BRCA1 *gene accounts for 45 percent of inherited breast cancer and more than 90 percent of inherited breast and ovarian cancer [3], and both genes combined account for only 25 percent of familial risk [7].

 The spectrum of mutations in these genes varies between populations, with some showing a high frequency of unique mutations [8]. Many such alterations may be recurrent, often being identified in isolated populations as the results of a founder effect [5] and may be the basis of differences in cancer risk between populations [9]. Ashkenazi Jewish, Norwegian, Dutch, and Icelandic people have a higher rate of certain genetic alterations in* BRCA1 *[10].

### 1.1. BRCA1 and BRCA2 Screening in Asia

 Most of *BRCA1 *and* BRCA2 *research has been focused on the Caucasian populations; however, the allelic frequency of higher penetrance genes in the Asian population may be higher than that in Caucasian population [11]. Immigrant Asian women especially from South Asia who are settled in the West show high rates of these cancers. Risch et al. [12] reported greater frequencies of mutations among cases of ovarian cancer that were of Indo-Pakistani descent than among British or mixed northern- or western-European ethnicity in Ontario, Canada. 

 Although 60 percent of the world's population resides in the Asian continent, and the fact that the Chinese, Malays, and Indians are three major Asian haplogroups, genetic predisposition to hereditary diseases and the applicability of genetic testing in these diverse ethnic groups is still unclear [13]. These regions have high rates of both these cancers especially the developing South Asian countries.

### 1.2. BRCA1 and BRCA2 Screening in Pakistani Population

 Amongst the Asian countries, Pakistan has one of the highest rates of breast and ovarian cancer [14] with breast cancer being the most common and ovarian the third most common malignancy amongst Pakistani women [15] and the most common cancer of gynecological origin in Pakistan [16]. A nine-year study period at a tertiary care cancer institution in Karachi, Pakistan showed that breast cancer was the most common cancer in females accounting for 38.2 percent of total cancer cases, at rates almost highest in Asia and ovarian cancer accounting for 4.9 percent [17]. Incidence rates in this region are on such a rapid rise that it is necessary to check the contributory factors, genetic and nongenetic. An insight into the above data emphasizes the formulation of a *BRCA1 *and *BRCA2 *database for the Pakistani population.

## 2. Methodology

 Study conducted by authors was carried out at the Centre for Research in Applied and Experimental Medicine, National University of Science and Technology, Rawalpindi. This study focused on mutational screening of *BRCA1 *gene in diagnosed cases of both hereditary and sporadic breast and ovarian cancer. In course of *BRCA1 *gene screening, all exon-intron boundaries were sequenced.

 These patients were interviewed after reviewing the history of their disease. Questionnaire was filled out containing information of their personal, family, and disease history. Ethnicity of each candidate was noted with the aim of finding founder effect mutations clustered to a specific ethnic group. 

Organic phenol-chloroform extraction method [18] was used to extract the genome. Amplification of all the exon-intron boundaries of *BRCA1 *gene by polymerase chain reaction (PCR) was performed using the oligonucleotide primers designed from the intronic sequences of the gene. Purified products were subjected to DNA sequencing by Automated Genetic Analyzer, Beckman Coulter CEQ8000, Genetic Analysis System. Alignment and mutational analysis was done using Bioedit software [19]. The sequencing chromatograms of the affected individuals were compared with the corresponding control full length gene sequences from UCSC Genome Browser [20] database to identify the aberrant nucleotide base pair change. Ensembl [21] database was used to recheck the gene sequence for cDNA and mRNA information. 

 Previously, four Pakistani studies depicted the mutational spectrum of these two genes. Two major pioneering investigations were accomplished by Liede et al. [22] at the National Cancer Institute, Karachi and Jinnah Hospital, Lahore and by Rashid et al. [14] at the Shaukat Khanum Memorial Hospital, Lahore. A comparatively small study focusing on mutational analysis of *BRCA1 *gene was performed by Malik et al. [23] at the COMSATS Institute of Information Technology, Islamabad. 

 Liede et al. [22] conducted a case-control study of 341 case subjects with breast cancer, 120 case subjects with ovarian cancer, and 200 female control subjects. Human genomic DNA was isolated from peripheral blood. Exon- intron boundaries were screened by protein-truncation testing (PTT). Direct sequencing was used for confirmation of all mutant bands detected by PTT.

 Rashid et al. [14] selected patients from 176 breast and/or ovarian cancer families who were diagnosed with invasive breast or epithelial ovarian cancer. Genomic DNA was extracted from blood samples. The entire coding regions of the *BRCA1* and *BRCA2* genes were screened using single strand conformational polymorphism (SSCP) analysis, denaturing high pressure liquid chromatography (DHPLC) analysis, and the protein truncation (PTT) assay [14]. Automated DNA Sequencing was performed for each sample revealing variants by SSCP, DHPLC, or PTT.

 150 cases of unilateral breast cancer patients were selected by Malik et al. [23]. After DNA extraction, Single strand conformational polymorphism (SSCP) was done for exons of *BRCA1,* and sequence analysis was performed for putative sequence variant. 

## 3. Results

**Figure 1:**
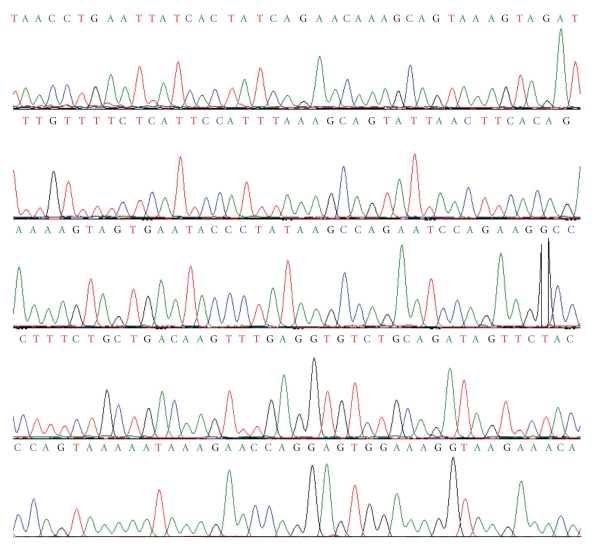
A representative chromatogram generated by automated genetic analyzer, Beckman Coulter CEQ TM, 8000 sequencing of Ex 13 of *BRCA1 *gene from an affected patient. Region between the two arrows indicates the exonic sequence. Flanking region is intronic sequence.

The results of samples recorded on BIORAD gel documentation system showed good yield and amplification of the DNA with the primers. The chromatograms of *BRCA1 *gene from affected patients (Figure 1) after mutational screening of the exons in all the samples of our study group revealed no pathogenic sequence variant correlating with breast or ovarian cancer pathogenesis. This is suggestive of the need to focus on the role of other high- or low-penetrance genes in breast and ovarian cancer predisposition in Pakistani population.

 By adding present and previous results regarding both *BRCA1* and *BRCA2* genes, a database with new percentage prevalence was formed. The role of *BRCA1* and *BRCA2* in the pathogenesis of breast and ovarian cancer in Pakistan was noted, and the most prevalent Pakistani mutations were highlighted. 

### 3.1. Percentage of BRCA1 BRCA2 Mutations in Breast and Ovarian Cancer Patients in the Pakistani Population

**Figure 2:**
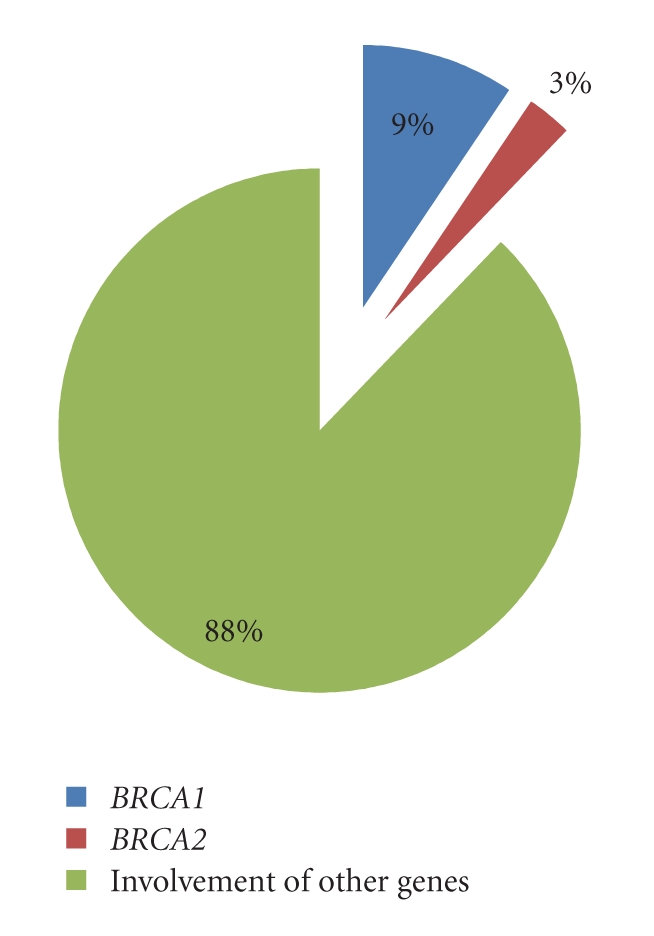
Genes involved in pathogenesis of breast and ovarian cancer in Pakistani population.

 Liede et al. [22] identified 42 sequence variants harboring 31 *BRCA1* mutations and 11 *BRCA2* variants in their study group of 341 women with breast cancer and 120 women with ovarian cancer. Rashid et al. [14] studied 176 Pakistani breast and ovarian cancer patients selected on family history and on age of diagnosis and identified a total of 30 sequence variants and among them 23 deleterious mutations in *BRCA1 *and 7 mutations *BRCA2 *gene. Malik et al. [23] detected a total of 6 variants in their samples (Tables 1 and 2). Taking all studies together, a total of 643 probands have been ascertained. Mutations in *BRCA1 *and *BRCA2* were found, respectively, in nine and three percent of breast and ovarian cancer patients (Figure 2). These results are authenticated by Risch et al. [12] who reported a high frequency of *BRCA1* mutations among cases of ovarian cancer that were of Indo-Pakistani descent (14%) along with Jewish (21%) and Italian (17%) ancestry in a population-based study of 649 cases of ovarian cancer in Ontario, Canada. These prevalence percentages highlight the significance of this review relevant to the Pakistani population. The remaining 88 percent of breast and ovarian cancers can be attributed to the involvement of other genes such as tumor protein (*TP53)* [MIM 191170], phosphatase and tension homolog (*PTEN)* [MIM 601728], checkpoint kinase 2 (*CHEK2)* [MIM 604373], and estrogen Receptor 1* (ESR) *[MIM 133430]. 

### 3.2. Most Prevalent BRCA1 and BRCA2 Mutations in the Pakistani Population

Twenty one distinct *BRCA1 *mutations were observed by Liede et al. [22] which are mostly insertions, deletions, or point mutations and exon 11, the largest exon of the gene, was mainly found to be the disease causing region. One intronic variant in exon 14 (IVS14-1G→A) was also noted (Table 1). Ten distinct types of *BRCA2 *variants were detected by these researchers, 9 in exon 11, and only one in exon 22, the 9140delA mutation in a Muhajir breast cancer patient (Table 2). The *BRCA2* 3337C→T mutation was found in two patients. Five *BRCA1 *mutations (2080insA, 3889delAG, 4184del4, 4284delGA, and IVS14-1ArG) and one *BRCA2 *mutation (3337C→T) were identified in multiple unrelated case subjects and represented candidate founder mutations. Rashid et al. [14] reported a total of twenty-two distinct mutations, 15 distinct variants in *BRCA1, *and 7 distinct variants in* BRCA2*. Among these were 12 frame shift mutations, 8 nonsense mutations, and 2 splice-site mutations (Tables 1 and 2). The most commonly observed *BRCA1* mutation was the 4627C→A, nonsense mutation identified in 5 families. Four mutations, 185delAG, 185insA, 4627C→A, and 5622 C→T, were recurrent; these accounted for 52 percent of all identified *BRCA1 *mutations. Malik et al. [23] observed two types of mutations in *BRCA1*, one insertion and one nonsense variant (Tables 1 and 2). Mutational analysis of all these studies emphasizes the justification of genetic testing for predisposing *BRCA1 *germ line mutations for any Pakistani family with multiple female breast and/or ovarian cancer cases [14].

**Figure 3:**
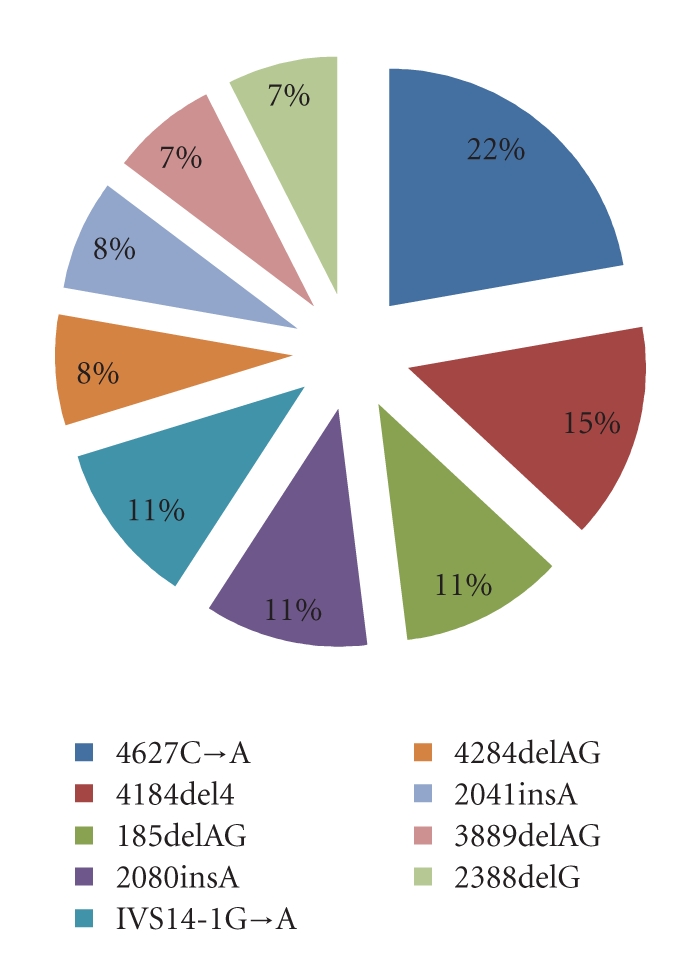
Most prevalent recurrent *BRCA1* mutations in Pakistani population.

**Figure 4:**
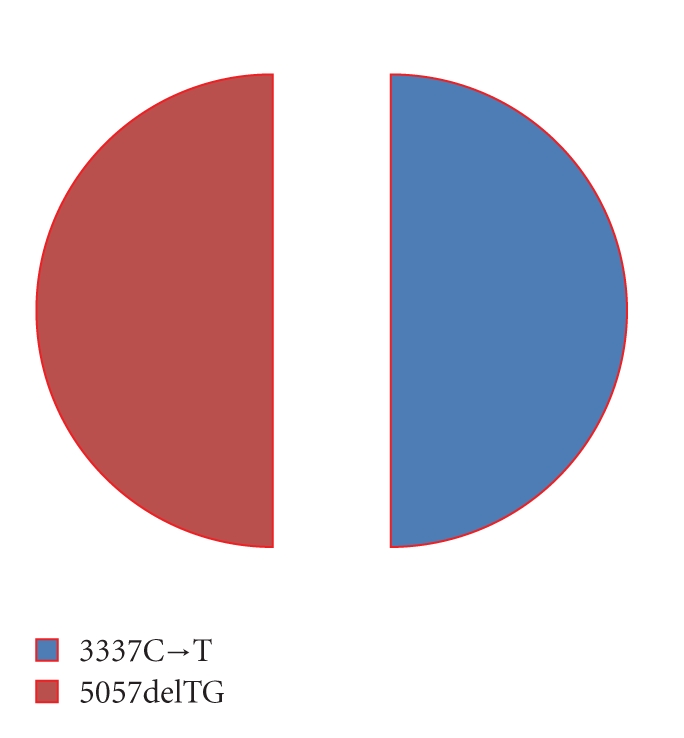
Most prevalent recurrent *BRCA2* mutations in Pakistani population.

In total,* BRCA1 *variant 4627C→A was observed in 22 percent of the probands showing recurrent mutations and features as the most prevalent *BRCA1* mutation in Pakistani population (Figure 3), while 15 percent of these patients showed the 4184del4 *BRCA1* variant which appears to be the second most prevalent mutation in Pakistani population (Figure 4).* BRCA2* mutational analysis revealed 3337C→T and 5057delTG to be equally prevalent (50 percent each) in the study participants showing recurrent *BRCA2* variants.

### 3.3. BRCA1 and BRCA2 Mutation Prevalence in Different Ethnic Groups of Pakistan

Pakistan has a pivotal location on the map of Asia, being at the crossroads of South Asia, the Middle East, and Central Asia. Pakistani population has a very rich anthrogeneological background owing to successive waves of invasions and adaptation of different haplogroups including Persians, Aryans, Mongols, Sikhs, Arabs, Greeks, Turks, and the British. Massive migrations from India in 1947 and more recently from Afghanistan have contributed to further diversifying the Pakistani population. These different populations are settled in four provinces of Pakistan. Punjabis comprise the largest ethnic group in the country at 44.15 percent, while other important ethnic groups include Pashtuns (15.42 percent), Sindhis (14.1 percent), Seraikis (10.53 percent), Muhajirs (7.57 percent), Balouchis (3.57 percent), and others (4.66 percent) [24]. The diverse ethnic blend concentrated in this region, especially in urban regions, contributes greatly to the genetic variability for inheritance of breast and ovarian cancer. 

The two major studies of Liede et al. [22] and Rashid et al. [14] included ethnicity as a vital determinant of mutational screening. In the 54 *BRCA1* mutations of both of these studies, 31 (57%) were found in the Punjabi ethnic group. Amongst the 18 *BRCA2* variants of both these studies, 6 (33%) were of Punjabi origin. Hence, the Punjabi ethnic group showed maximum sequence variants in the *BRCA *gene variants (Table 3).

### 3.4. Candidate Founder Mutations of BRCA1 and BRCA2 for the Pakistani Population

Twelve of the 21 *BRCA1 *mutations (57 percent) and 8 of the 10 *BRCA2 *mutations (80 percent) detected by Liede et al. [22] were unique to the Pakistani population [22], while Rashid et al. [14] detected ten mutations (33 percent) which were unique to the Pakistani population, which is comprised of 5 *BRCA1 *mutations (33 percent) and 5 *BRCA2 *mutations (71 percent). The finding of thirteen recurrent *BRCA1 *and two recurrent* BRCA2 *mutations in some members of the Pakistani population could allow very economical screening for such mutations in specific ethnic groups in the country which could be of great benefit to public health measures.

The most prevalent *BRCA1* mutation of both studies combined, 4627C→A, was found in six Punjabi patients increasing the likelihood of finding this variant in Punjabi ethnicity. Four probands showed the 4184del4 *BRCA1 *variant out of which three were Punjabi and one Sindhi. All the four carriers of the *BRCA1 *185insA and the two carriers of the *BRCA1 *5622 C→T were also of Punjabi ethnicity. This predisposition of the Punjabis to maximum *BRCA* variants can lead to focusing on the specific sequence variants of this ethnic group. All the carriers of the* BRCA1 *2080insA mutation were of Pathan ethnicity. Rashid et al. [14] also found 185delAG in two non-Jewish unrelated Pakistani carriers of Pathan ethnicity, whose ancestors originated from the same geographic region in the North-West Frontier Province of Pakistan. These two mutations can be the focus of candidate founder mutations in this ethnic group. The two cases with the *BRCA1 *4284delAG mutation belonged to the Muhajir ethnic group, again signifying the importance of focusing on this mutation in this particular ethnic group. 

In *BRCA2 *screening, Liede et al. [22] found the recurrent 3337C→T variant in two Memon breast cancer patients pointing towards the significance of finding this mutation in other Memon patients as well. 5057delTG *BRCA2 *mutation was a finding of both studies; Rashid et al. [14] detected this mutation in a Punjabi patient, while Liede et al. [22] detected it in a minority ethnic group, a Parsi ovarian cancer patient. 

### 3.5. Possible Management Strategies

 Ever since the discovery of breast and ovarian cancer genes, many advances in clinical research have been made which provide the rationale for moving genetic testing of these genes into clinical practice [25]. *BRCA1* and *BRCA2,* like other genes, have not only served as molecular markers for hereditary breast cancer risk screening but also become important indicators for breast cancer prevention, treatment, and prognosis [26]. Genetic testing is gaining acceptance worldwide and has been established throughout North America and much of Europe. Genetic counseling, especially in country like Pakistan having one of the highest rates of consanguinity [27, 28], is practiced by many health professionals; however, its expansion into the health policy and training regimes of health care providers is needed. The awareness, however, of genetic testing as a tool for preventive and treatment-oriented management of these tumors is limited. Formulation of *BRCA* mutational database can serve as a cost-effective tool to identify individuals at high risk such as unaffected carrier relatives who have a defective allele that can be transferred to offspring. Such tests are strategized on the basis of most prevalent mutations in a particular population/ethnic group that can save both time and money. Till then the most effective options for breast cancer risk reduction include prophylactic bilateral mastectomy [29] prophylactic oophorectomy, and oral administration of Tamoxifen [26] in some countries. If genetic testing for these genes becomes streamlined, these prophylactic procedures and other preventive strategies will be warranted to become routine for mutation carriers along with vigilant medical checkups, especially in an economically challenged country like Pakistan.

## 4. Conclusions

The need for larger collaborative studies between medical professionals and molecular biologists cannot be emphasized enough. This is necessary for gaining further insight into mutational spectra and ethnic distribution of different types of mutations, in a search for founder effect mutations, which can become a part of cancer screening policies in Pakistan. The high prevalence of these cancers and the presence of recurrent mutations of these genes in the Pakistani population, especially the observation of a high percentage of *BRCA1* variants in ovarian cancer cases, emphasize the need for improving genetic counseling strategies and make genetic testing a part of screening policies. The more work done on the genomics of this disease with relevance to the Pakistani population, the closer a genetic cure targeted for this specific population can be found. 

##  Conflict of Interests

The authors declare that they have no Conflict of interests.

##  Authors' Contributions

A. Farooq contributed in the design of study experimentation; A. K. Naveed is the Supervisor of study; Z. Azeem assisted in experimentation and data compilation; T. Ahmed assisted in data compilation.

## Figures and Tables

**Table 1:** Mutational spectrum of *BRCA1* gene in Pakistani population.

No	Exon no.	Point of mutation	Mutation	Ethnicity	Reference
*Deletion*					

*Type*					

1	2	185delAG	frameshift and truncation	1 Punjabi 2 Pathan	Liede et al. [22] Rashid et al. [14]
2	11	1616delAAAT	frameshift and truncation	1 Muhajir	Liede et al. [22]
3	12	4284delAG	frameshift and truncation	2 Muhajir	Liede et al. [22]
4	11	4184del4	frameshift and truncation	3 Punjabi 1 Sindhi	Liede et al. [22]
5	11	1476delG	frameshift and truncation	1 Punjabi	Liede et al. [22]
6	11	3889delAG	frameshift and truncation	2 Punjabi	Liede et al. [22]
7	11	2388delG	frameshift and truncation	2 Muhajir	Liede et al. [22]; Rashid et al. [14]
8	11	894delG	frameshift and truncation	1 Muhajir	Liede et al. [22]
9	11	1956delA	frameshift and truncation	1 Punjabi	Liede et al. [22]
10	11	1127delA	frameshift and truncation	1 Punjabi	Liede et al. [22]
11	11	2266delG	frameshift and truncation	1 Punjabi	Liede et al. [22]
12	7	550delA	frameshift and truncation	1 Multiracial	Rashid et al. [14]
13	8	589delCT	frameshift and truncation	1 Punjabi	Rashid et al. [14]
14	17	5149delCTAA	frameshift and truncation	1 Punjabi	Rashid et al. [14]
15	11	1013delTG	frameshift and truncation	1 Muhajir	Liede et al. [22]

*Insertion*					

*Type *					

16	11	2080insA	frameshift and truncation	3 Pathan	Liede et al. [22]
17	11	2041insA	frameshift and truncation	2 Punjabi	Liede et al. [22]
18	2	185insA	frameshift and truncation	4 Punjabi	Liede et al. [22]; Rashid et al. [14]
19	11	1770insT	frameshift and truncation	1 Balouchi	Liede et al. [22]
20	11	3812insT	frameshift and truncation	1 Multiracial	Rashid et al. [14]
21	20	5376insA	frameshift and truncation	1 Multiracial	Rashid et al. [14]
22	13	4356insA	frameshift and truncation	3 Not specified	Malik et al. [23]

*Nonsense*					

*Protein changed*					

23	15	4627C→A, S1503X	Stop 1503	6 Punjabi	Liede et al. [22]; Rashid et al. [14]
24	11	1590 C→T, Q491X	Gln to stop	1 Punjabi	Rashid et al. [14]
25	11	1731C→T, Q531X	Gln to stop	1 Multiracial	Rashid et al. [14]
26	12	4302C→T, Q1395X	Gln to stop	1 Punjabi	Rashid et al. [14]
27	24	5622C→T, R1835X	Arg to stop	2 Punjabi	Rashid et al. [14]
28	11	1912T→G	Gln to stop	1 Muhajir	Liede et al. [22]
*Missense*					

*Protein changed*					

29	13	4305	Serine changed	3 Not specified	Malik et al. [23]
30	11	3405C→T	C→T	1 Muhajir	Liede et al. [22]
31	11	2722C→G	C→G	1 Kashmiri	Liede et al. [22]

*Splice site*					

32	Intron 14	IVS14-1G→A		2 Punjabi 1 Pathan	Liede et al. [22]
33	Intron 4	IVS4-1G→T		1 Punjabi	Rashid et al. [14]
34	Intron 20	IVS20-1G→C		1 Multiracial	Rashid et al. [14]

**Table 2:** Mutational spectrum of *BRCA2* gene in Pakistani population.

No	Exon no.	Point of mutation	Mutation	Ethnicity	Reference
*Type of mutation*					

*Deletion*					

1	22	9140delA	frameshift and truncation	1 Muhajir	Liede et al. [22]
2	11	3913delG	frameshift and truncation	1 Sindhi	Liede et al. [22]
3	11	5950delCT	frameshift and truncation	1 Punjabi	Liede et al. [22]
4	11	6696delTC	frameshift and truncation	1 Punjabi	Liede et al. [22]
5	11	2674delG	frameshift and truncation	1 Muhajir	Liede et al. [22]
6	11	5057delTG	frameshift and truncation	1 Punjabi 1 Parsi	Liede et al. [22]; Rashid et al. [14]
7	11	3179delA	frameshift and truncation	1 Muhajir	Liede et al. [22]
8	10	1993delAA	frameshift and truncation	1 Multiracial	Rashid et al. [14]
9	11	4052delTAGA	frameshift and truncation	1 Multiracial	Rashid et al. [14]
10	25	9658delT	frameshift and truncation	1 Multiracial	Rashid et al. [14]

*Insertion*					

11	11	5302insA	frameshift and truncation	1 Punjabi	Liede et al. [22]
12	11	6679insAA	frameshift and truncation	1 Punjabi	Liede et al. [22]

*Nonsense*					

*Protein changed*					

13	11	3337C→T	Gln to stop	2 Memon	Liede et al. [22]
14	10	2083C > T, Q619X	Gln to stop	1 Punjabi	Rashid et al. [14]
15	11	3218T > G, L992X	Leu to stop	1 Multiracial	Rashid et al. [14]
16	11	5962G > T, E1912X	Glu to stop	1 Multiracial	Rashid et al. [14]

**Table 3:** Ethnic distribution of *BRCA* carriers in Pakistani population.

*BRCA1* mutations		*BRCA2* mutations	
Ethnicity	%	Ethnicity	%
Punjabi	57	Punjabi	33
Muhajir	17	Multiracial	28
Pathan	13	Muhajir	17
Sindhi	1.8	Memon	11
Balouchi	1.8	Parsi	5.5
Kashmiri	1.8	Sindhi	5.5
Multiracial	7.4		
